# Methanol electro oxidation on Ni–Pt–CrO/CNFs composite: morphology, structural, and electrochemical characterization

**DOI:** 10.1038/s41598-023-31940-x

**Published:** 2023-03-24

**Authors:** E. E. Abdel-Hady, Ahmed Gamal, Hany Hamdy, Mohamed Shaban, M. O. Abdel-Hamed, Mahmoud A. Mohammed, Wael M. Mohammed

**Affiliations:** 1 Physics Department, Faculty of Science, Minia University, P.O. Box 61519, Minia, Egypt; 2 Nanophotonics and Applications (NPA) Lab, Department of Physics, Faculty of Science, Beni-Suef University, Beni-Suef, 62514 Egypt; 3 Physics Department, Faculty of Science, Islamic University of Madinah, P.O. Box 170, Madinah, 42351 Saudi Arabia

**Keywords:** Chemistry, Energy science and technology, Materials science, Nanoscience and technology, Physics

## Abstract

In this work, prepared nanoparticle samples of Ni_1-x_Cr_x_ with a fixed ratio of platinum (3%) were synthesized and loaded onto carbon nanofibers, which were produced by an electrospinning technique and carbonized at 900 °C for 7 h in an argon atmosphere. A variety of analysis techniques were applied to examine the stoichiometry, structure, surface morphology, and electrochemical activity. The carbonization process produces carbon nanofibers decorated with metal nanoparticles. Typical fibre diameters are 250–520 nm. The fibre morphologies of the treated samples don’t exhibit any overt alterations. A study of the samples’ methanol electrocatalytic capabilities was conducted. Cyclic voltammetry, chronoamperometry, and electrochemical impedance measurements were used to investigate catalytic performance and electrode stability as a function of electrolyte concentration, scan rate, and reaction time. The electrooxidation reaction’s activation energy is increased, and the electrode’s stability is increased, when Cr is added to Ni. In sample C3, the maximum current density (JPE) was 170.3 mA/cm^2^ at 0.8 V with an onset potential of 0.352 V. Utilizing our electrocatalysts, the electrooxidation of methanol involves a mix of kinetic and diffusion control limiting reactions. This study has shown how to fabricate a powerful Ni–Pt–Cr-based methanol electrooxidation catalyst using a novel approach.

## Introduction

Current research in the area of sustainable and renewable energy is concentrating on the enhancement of fuel cells in order to address the problem of the depletion of fossil resources. Fuel cells have attracted a lot of attention due to their benefits, including their high efficiency and almost low emissions. On the other hand, fuel cells offer an effective and healthy method of converting energy. Additionally, for sustainable development and energy security, it works well with modern energy sources like hydrogen and renewable energy sources. As a result, they are seen as the energy conversion technologies of the future. Fuel cells’ static nature also makes them noiseless and vibration-free, and their intrinsic flexibility enables simple assembly and a wide range of uses in portable, fixed, and transportation power generation. In essence, fuel cells offer the cleanest, most flexible, and most effective method of converting chemical energy into electrical energy, with high power density, simple scaling, and low operating temperatures^1^.

However, due to the high cost and limited supply of Pt catalysts, there is increasing interest in developing free or low-platinum group metal catalysts for use in Proton-exchange membrane fuel cell (PEMFCs)^2^. Because of their unique qualities, precious metallic nanoparticles (NPs) and materials made of carbon are frequently utilized in the catalytic industries. For instance, Pt is used in cars to transform toxic exhaust gases, including hydrocarbons, NOx, and CO, into CO_2_, N_2_, and H_2_O^3^. Additionally, Pt is frequently used as a catalyst in fuel cells. It is said that the functioning of fuel cells is limited by the sluggish oxygen reduction (OR) and its slow kinetics on Pt. To improve the activity and stability of Pt catalysts in the negative electrode, several teams have conducted a number of studies^4,5^. As a co-catalyst, tin is extensively used to improve the electrochemical oxidation activity of Pt towards methanol oxidation Pt/Fe, Pt/Co, Pt/Ni, and Pt/Cr are a few of the different Pt alloy-based catalysts that have been created Due to their low overpotential and high catalytic activity, in PEMFCs^6–9^. Pt-alloys comprising various transition metals, including Ni, Cr, Co, and others, have demonstrated higher ORR electrocatalytic activity in PEMFCs as compared to pure Pt^10,11^. This development is possible and can be attributed to several things, including a decline in the oxidation state of Pt, the creation of novel electronic structures with higher energy, and the inhibition of Pt oxide production^12,13^. Many efforts have been focused on the development of low- and non-precious metal catalysts to overcome the technical barriers^14,15^. Nickel based materials are among the most promising candidates because of their good chemical stability, electrical properties, and ability to remove intermediate COad in alkaline media [^16,17^]. Ni demonstrated encouraging electrochemical oxidation activity for both methanol and urea among the examined transition metals^18,19^. Several methods have been used to increase Ni's activity in order to increase its catalytic activity and durability, including increasing its surface area and integrating non-precious metallic elements, complexes, and oxides. The methanol oxidation reaction was examined using Ni–Co nanoparticles^20^, Ni/TiO_2_ nanotubes^21^, Ni–Cd coated graphite^22^, Ni–Cu alloy^23^, and Ni–Cr nanooxides^24^. These materials showed improved electrocatalytic activity and stability (MOR).

Due to the high energy density of methanol fuels, ease of storage, and low pollution, direct methanol fuel cells (DMFCs) have attracted the most interest, and, as a consequence, the creation of low-cost, high-activity anodes for DMFCs has emerged as a critical area of research. To replace Pt-based materials, several attempts are being made to create efficient non-precious electrocatalysts. Nickel metal and its alloys have received a lot of attention as viable candidates for methanol oxidation due to their low cost and high activity. It has been established that methanol oxidation involves both electrochemical processes and adsorption on the surface of the anode^25–27^. So, carbon has been incorporated into various recently reported electrocatalytic materials, not only for DMFCs but similarly for other types of fuel cells, to take advantage of its adsorption potential. By absorbing the methanol molecules, carbon serves the main purpose of attracting them to the catalytic material.

In this study, a low-cost supporting nanofiber was used to build a particulate nanocomposite of Ni, Cr, and a set amount of Pt (3%) for mass production of inexpensive nano electrocatalysts on the way to the creation of marketable fuel cells. The synthesis and characterization of Ni and Cr-based Pt-replacement electrocatalysts are the goals of this work. Here, NiPtCrO/CNF nanoparticles are incorporated into carbon nanofibers, which are generated, characterised, and investigated as an electrocatalyst for methanol oxidation in an alkaline medium (KOH). To the best of our knowledge, no previous work has been done on this system. The impact of the Ni/Cr ratio on the morphologies, chemical composition, structures, and electrooxidation performance of electrocatalysts was examined using scanning electron microscopy (SEM), transmission electron microscopy (TEM), EDX mapping, and X-ray diffraction (XRD). The electrocatalytic assets of the fabricated catalysts are inspected for the electrooxidation of methanol. On the effectiveness of the fabricated catalysts, the impacts of electrolyte content, reaction time, and SR are investigated. The performance and stability of the electrodes are further evaluated using techniques such as cyclic voltammetry, chronoamperometry, and electrochemical impedance spectroscopy (EIS). Ni–Pt–Cr ternary nanoparticles are likely to be useful as catalysts in fuel cell applications.

## Experimental

NiAc (98%) is a nickel (II) acetate tetrahydrate, chromium nitrate Cr (NO_3_)_3_, 98%, and H_2_PtCl_6_ in various ratios are added to a 10% PVA solution in water to create a set of electrocatalysts. Typically, NiAc, CrNO_3_, and H_2_PtCl_6_ were combined with the solution of PVA to form a mixture containing PVA and 20% (Ni, Cr/Pt) of different atomic percentages of individual metals, according to Table 1.

**Table 1:** The electro-catalysts’ metal mixture with a 20% total metal loading.

Catalyst name	Cr%	Pt%	Ni%
C1	0	3	17
C2	3	3	14
C3	6	3	11

The mixture was agitated overnight to obtain a clear liquid at ambient temperature. For the electrospinning process, the clear solutions were put into a 20 ml container. A voltage of 20 kV, a working distance of 15 cm, and a flow rate of 0.3 mL/h were the ideal electrospinning settings. A fibrous web of nanofibers formed on the metal plate's surface. The electrospun mates were dehydrated in a vacuum for 24 h at 80 °C before being carbonized for 7 h in an argon atmosphere at 900 °C with a heating rate of 3 °C/min and a holding time of 2 h. The schematic diagram shown in Fig. S1 exhibits the procedures, starting with preparation and ending with scanning the samples.

### Preparation of GC electrode

Once the nanofiber has been carbonized, it should be ground up and weighed. Next, combine 50 μL of Nafion (D-521 dispersion, 5% w/w in water and 1-propanol, $\ge$ 0.92 meq/g exchange—Alfa Aesar), with 400 μL isopropanol, and set the mixture in an ultrasonic for 30 min. After 10 min of oven drying, the sample was precipitated on an electrode before being analyzed by cyclic voltammetry in 1 M KOH.

### Characterization

SEM/EDX (Jeol JSM—I T 200) was utilized to analyze the morphology of the nanofibers’ surface and their stoichiometry. The measurements were done at the Central Laboratory for Microanalysis and Nanotechnology, Minia University, Egypt. The XRD spectroscopy (202,964—Panalytical Empryan) with Cu K (0.154 nm) radiation had been used to inspect the crystal structure of the fabricated catalysts. XRD pattern was studied at 0.05 step size over the range of 10°–100°. The XPS technique was used to obtain information on the chemical composition of the sample’s surface as well as the depth distribution of chemical species used as catalysts. An electrochemical analyzer (CHI660E series, Austin, TX, USA) connected to an electrochemical cell with three electrodes a reference electrode (RE) (Ag/AgCl; KCl solution of 4.0 M concentration), an auxiliary or counter electrode (CE) (platinum wire), and a working electrode (WE) represented by a glassy carbon electrode; GC, carried out the electrocatalytic activities of the electrodes for methanol electro-oxidation.

## Results and discussions

### Morphological features

**Figure 1:**
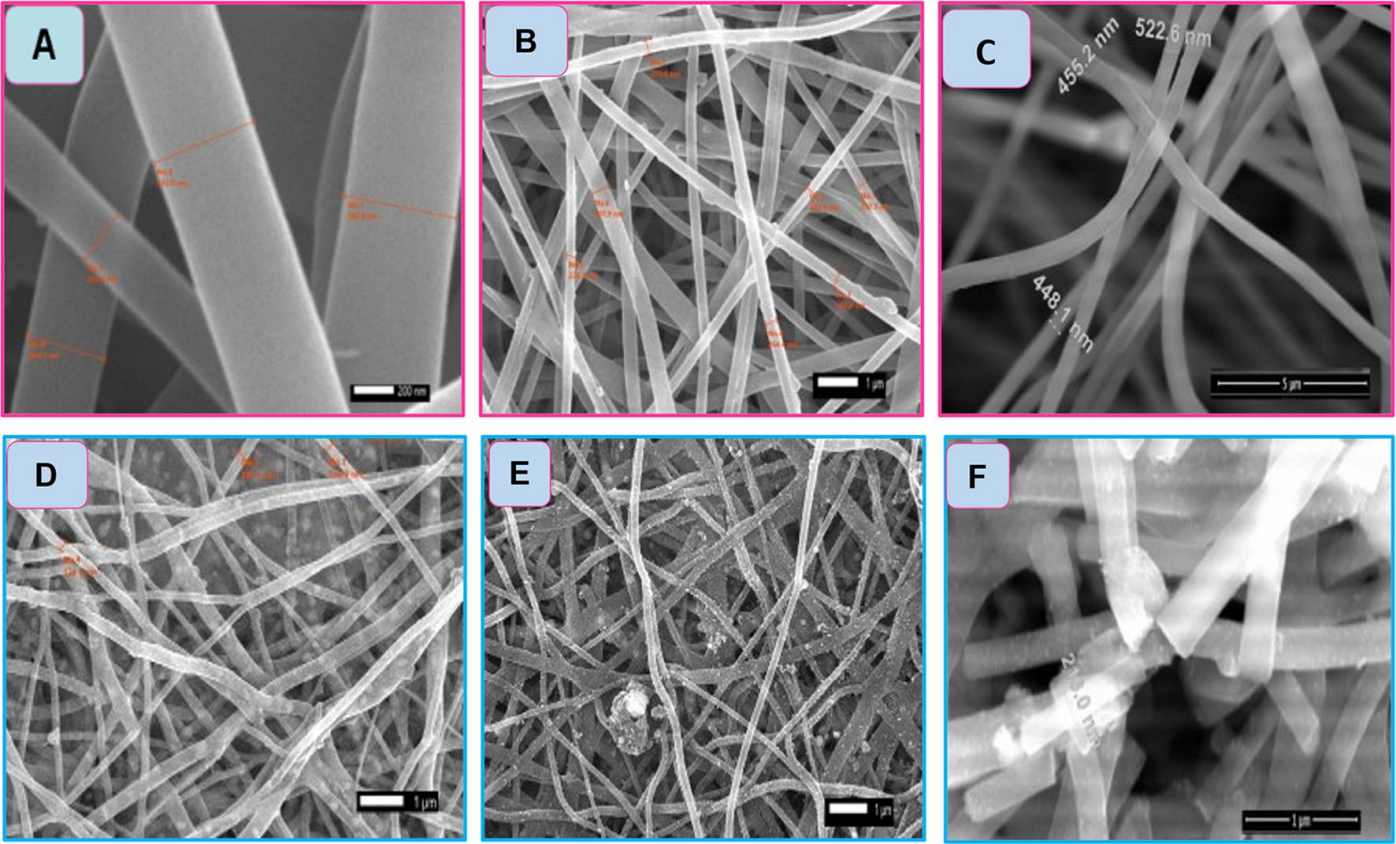
SEM images of (**A**) Ni_17_Pt_3%_/CNFs, (**B**) Ni_14_Pt_3_Cr_3_CNFs, (**C**) Ni_11_Pt_3%_Cr_6%_/CNFs before carbonization, (**D**) Ni_17_Pt_3%_/CNFs, (**E**) Ni_14_Pt_3_Cr_3%_/CNFs, and (**F**) Ni_11_Pt_3%_Cr_6%_/CNFs after carbonization.

**Figure 2:**
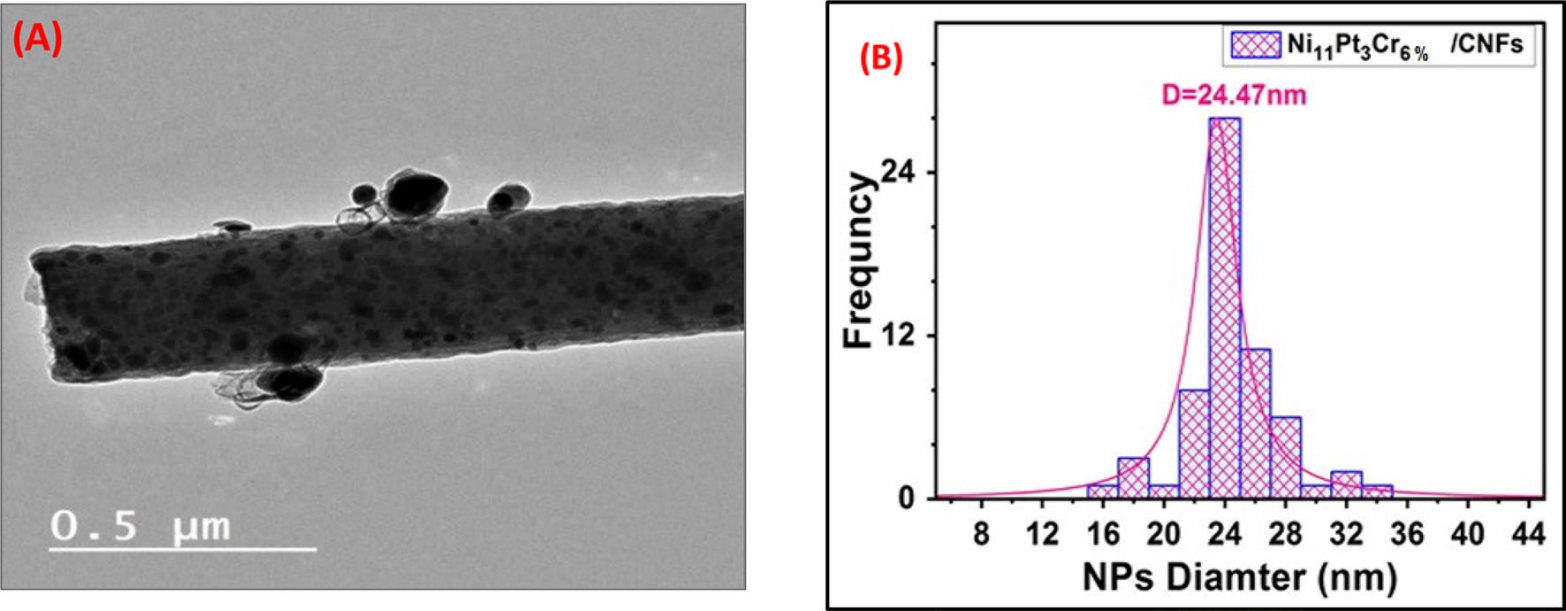
(**A**) TEM micrograph and (**B**) size distribution histogram of Ni_11_Pt_3_Cr_6%_/CNFs.

Figure 1A,B,C displays SEM images of PVA polymers loaded with Ni_17%-x_Cr_x_Pt_3%_ and different ratios of Cr_x_ (x = 0, 3, and 6%) before carbonization. The Electrospinning technique was utilized to magnificently synthesize PVA/NiAc-Cr(NO_3_)_3_-H_2_PtCl_6_ nanofiber. The great quality of all manufactured nanofibers is shown in the figure since they have good physical characteristics such as being continuous, uniform, and bead-free. The fibre morphologies of the produced samples did not significantly differ, and their diameter ranged from 250 to 520 nm. After being carbonized at 900 °C in an atmosphere with argon gas, the generated carbon nanofibers are visible in SEM pictures (Fig. 1D,E,F). The CNFs may effectively limit the metal nanoparticles (NPs) size and prevent the NPs from clumping together during the catalytic process^28,29^. It would be mentioned that the CNF network’s connected 3D architecture is regarded as a suitable support material because it creates expansive interfaces and interstices between the electrolyte and catalytically active areas, facilitating rapid electron transport and quick gas diffusion^30^. The SEM images in Fig. 1D,E,F show that following carbonization, metal nanoparticles (NPs) were successfully prepared and enclosed in CNFs. This can be qualified by the transition metal's capacity to attach to PVA’s hydroxyl groups before binding to the carbon following calcination^31^. Additionally, as revealed in the image, the calcination method of the CNFs reduced the fiber's width to run between 216 and 350 nm rather than having a discernible impact on the nanofibers' morphology. TEM is a technique for investigating the metal nanoparticles' size and their distribution on the (CNFs). Figure 2A shows a TEM image of sample C3 (Ni_11_Pt_3_Cr_6_%/CNFs). The TEM image showed that the Ni_11_Pt_3_Cr_6_% NPs was distributed almost homogeneously on the carbon fibers' surface. The average diameter of the nanoparticles was 24 nm, as displayed in Fig. 2B. To test the fiber quality of the samples after the oxidation process, SEM images have been captured after the oxidation as shown in Fig. S2. As can be seen from the figure, the samples' SEM pictures following oxidation reveal little to no change in fibre quality, which is indicative of the high durability of the produced catalysts.

### The compositional analysis (EDX)

**Figure 3:**
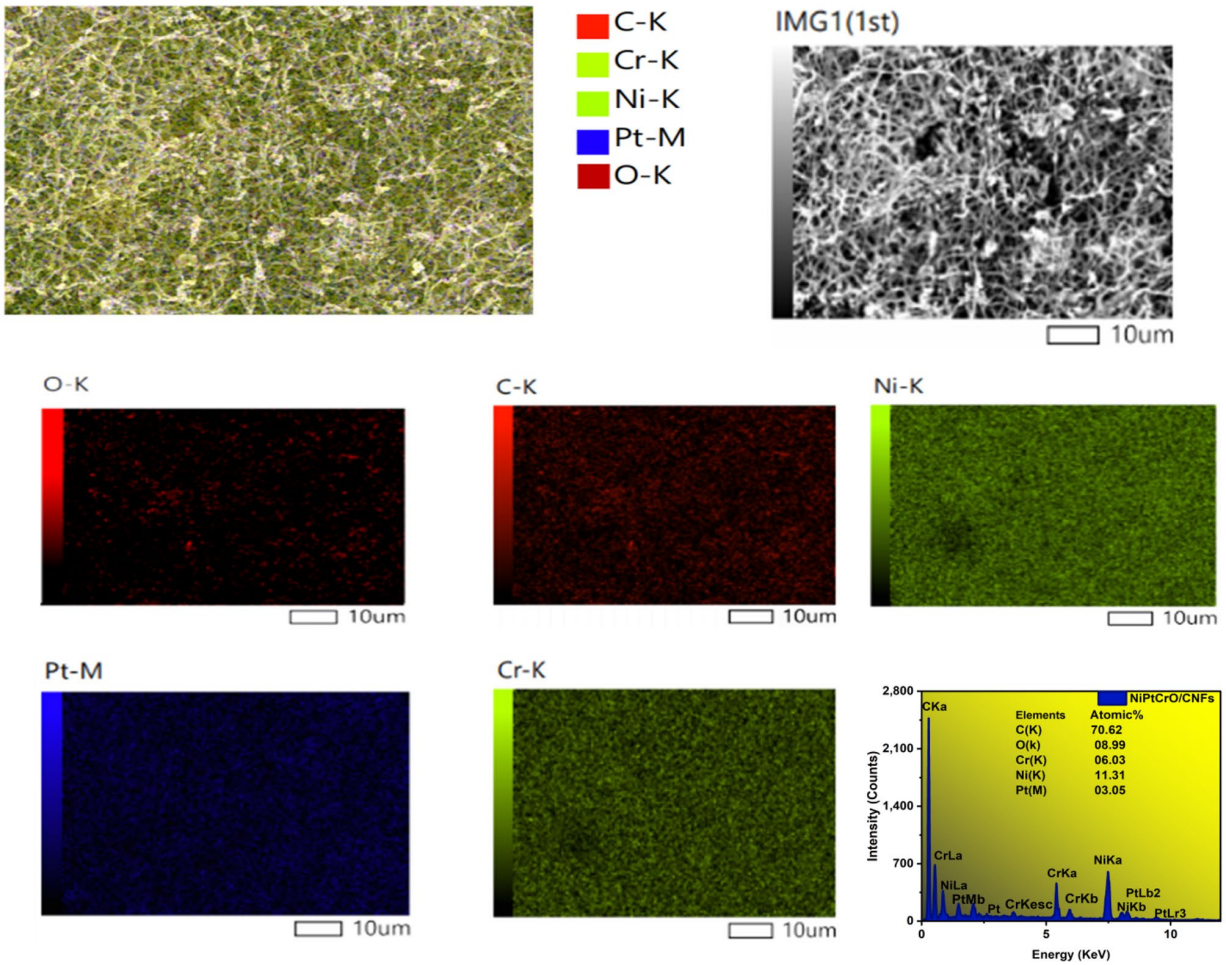
SEM matched with elemental mappings and EDX analyses for the sample Ni_11_Pt_3_Cr_6_%/CNFs.

For the sample C3, Fig. 3 displays the SEM image with EDX analysis and the related EDX mapping. It demonstrates that the C element was evenly and densely distributed throughout the whole sample as nanofibers. Furthermore, the elemental recording images (Fig. 3) showed that the shining NPs were composed of Ni, Pt, Cr, and O, with no other imported contaminants. This study validates the XRD findings by demonstrating the incorporation of Pt, Cr, and Ni into carbonised PVANFs. This study validates the XRD findings by demonstrating the incorporation of Pt, Cr, and Ni into carbonized PVANFs. The attraction of the transition metal to the hydroxyl groups in PVA and the resulting carbon content during carbonization allow this to happen. It is believed that using the suggested preparation method will result in carbon nanofibers that have undergone carbonization and have been embellished with metal nanoparticles. Through EDX examination, the sample C3’s Ni11 Pt3 Cr6% chemical composition was determined, and the elements' atomic proportions are also recorded in Fig. 3. Atomic percentages for C, Ni, Cr, Pt, and O are 70.62%, 11.31%, 6.03%, 3.05%, and 8.99%, respectively.

### X-ray photoelectron spectroscopy (XPS)

**Figure 4:**
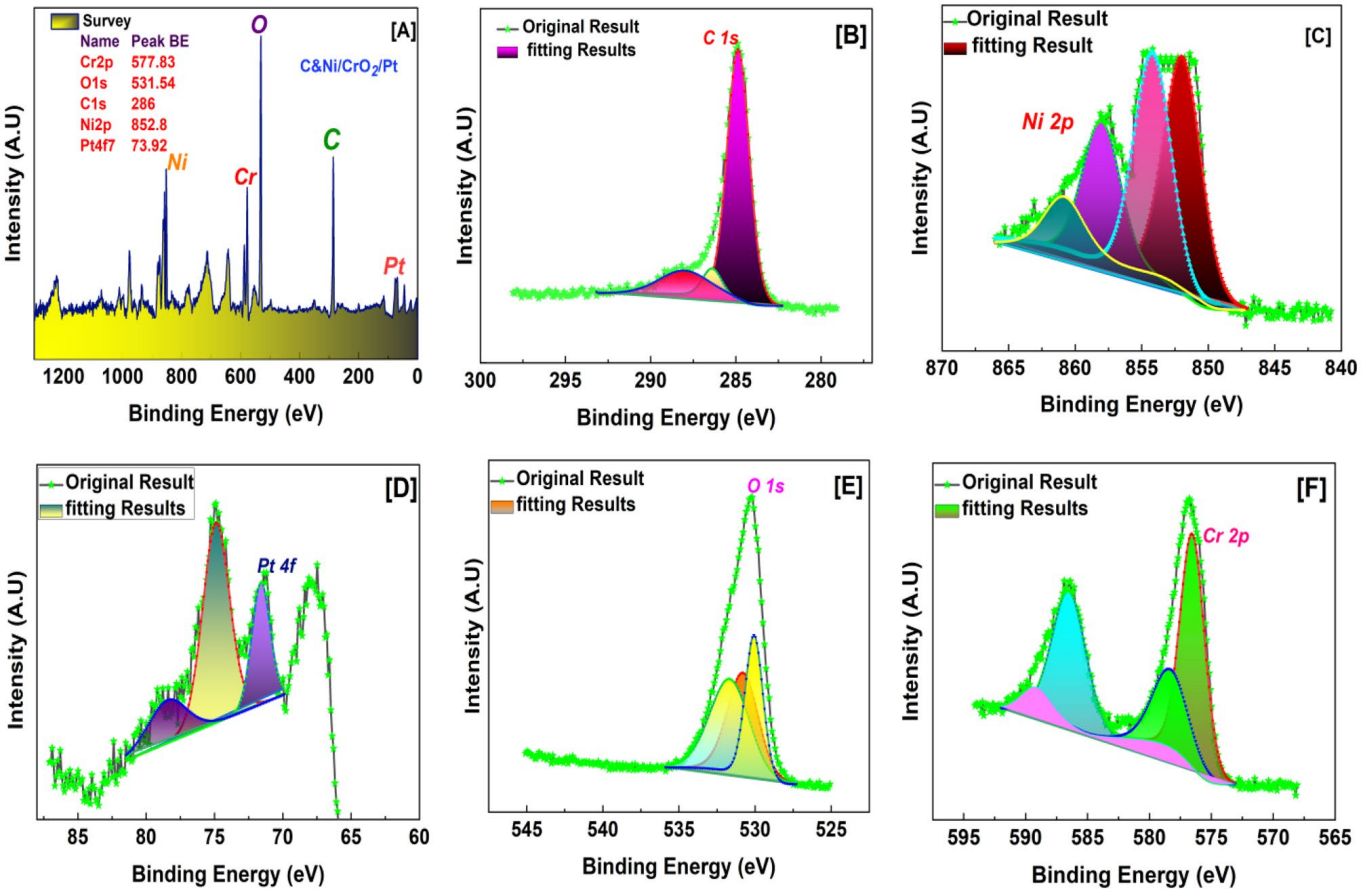
XPS analysis of the sample C3 (Ni_11_Pt_3_Cr_6_%/CNFs) and the detailed scan for C 1s, Ni 2p, Pt 4f., O 1s and Cr 2p.

X-ray photoelectron spectroscopy (XPS) was conducted to study the chemistry of the surface and the valence state of the incorporated transition metals. Figure 4A shows the XPS survey spectra of NiPtCrO alloy NPs/CNFs, which confirm the presence of Ni 2p, Pt 4f., C 1s, Cr 2p and O 1s. The C 1s XPS detailed spectrum, as shown in Fig. 4B, demonstrates the presence of one type of carbon species corresponding to the peak located at 284.86 eV. The high-resolution spectrum of Ni 2p is presented in Fig. 4C. Ni 2p_3/2_ can be assigned to the observed peaks at 852 eV and 853.8 eV, respectively^31,32^. Furthermore, satellite peaks of Ni 2p_3/2_ are seen at 858.1 and 861.1 eV, respectively, adjacent to the main peaks. The peak of Pt in the Pt 4f spectra (Fig. 4D) is at 71.6 and 74.8 at line spectra 4f. _7/2_ and 4f. _5/2_, respectively^33^.

In the Cr 2p spectra (Fig. 4F), the peaks of NiPtCrO/CNFs at 576.2 and 585.9 eV can be ascribed to Cr–O bonds of Cr 2p_3/2_ and 2p_1/2_ orbitals in Cr 2p^34,35^. The results support the XRD results for the crystalline Cr_2_O_3_. Finally, the oxygen spectrum O 1 s spectrum can be fitted into three peaks, as shown in Fig. 4E, which pertain to the Metal-O bond in Cr_2_O_3_ (Cr–O, 530.7 eV), and oxygen vacancies (O_V_, 530.06 eV).

### Structure characterization and phase evaluation (XRD)

**Figure 5:**
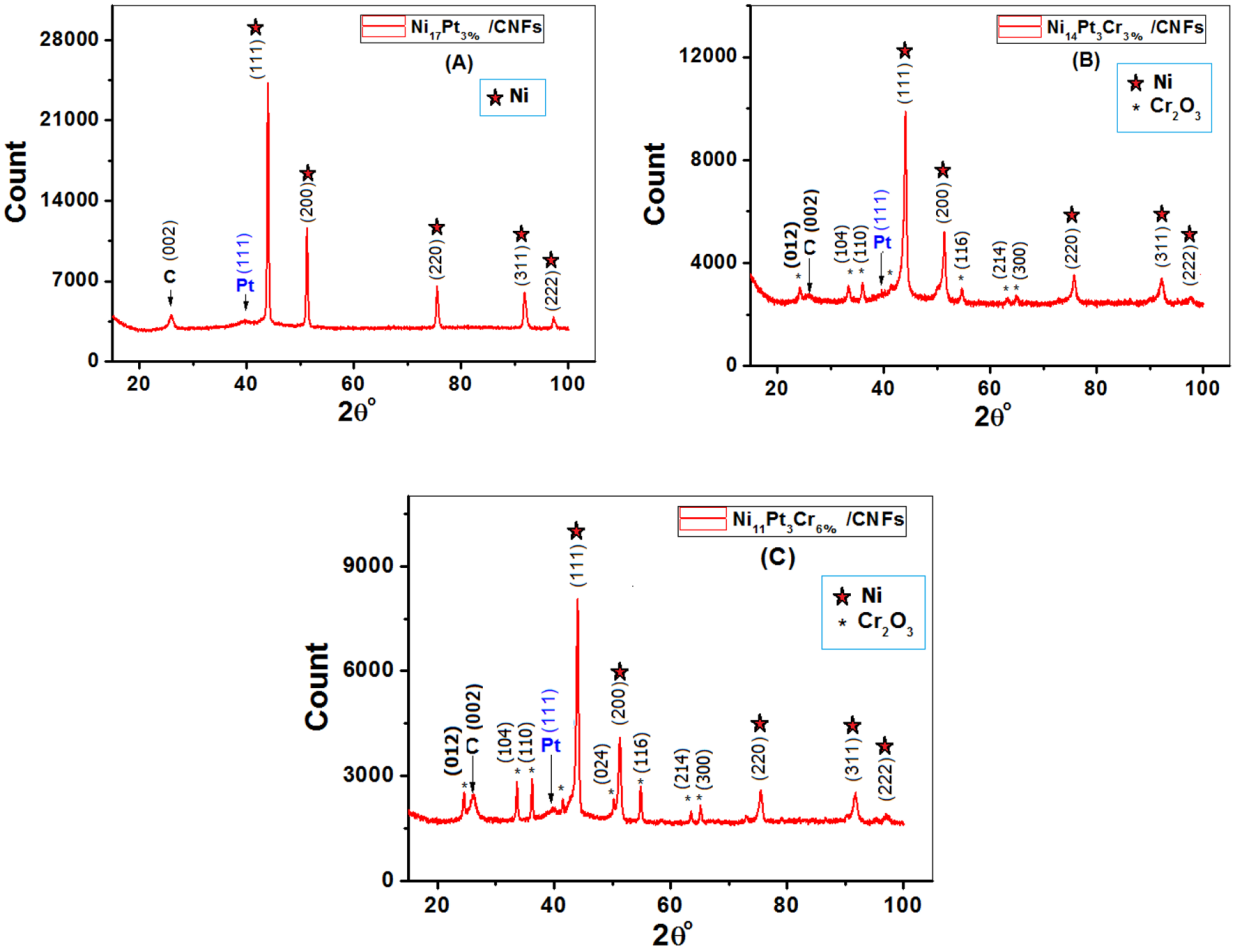
XRD patterns of CNFs/Ni_17−x_Cr_x_-Pt_3%_ nanoparticles [(**A**) x = 0, (**B**) x = 3 and (**C**) x = 6%].

The investigation of the crystal structure and content of the carbonized nanofibers can be done with high certainty using XRD analysis. Figure 5A,B,C shows that metallic NPs were produced in the NF matrix based on X-ray diffraction results. From Fig. 5, it is evident that five peaks (red stars) related to the (111), (200), (220), (311), and (222) Ni crystal planes can be detected near 44°, 51°, 76°, 92°, and 97° for Ni NPs in all samples, steady with an FCC nickel crystallite implanted in the surface of the NFs (JCPDS File No. 04–0850)^36,37^. The result of XRD displays sharp peaks that indicate high crystallinity nanoparticles. Referring to Pt, only one peak is seen at 2θ = 40°, which indicates the (111) Bragg’s plane of Pt’s FCC. The observed peak at 2θ ~ 26° indicates the formation of graphite-like carbon (002), (JCPDS; 41–1487)^38,39^. Since the CNFs were graphitic, the conductivity of the generated electrodes would increase^40^. The smallness of this peak’s intensity is due to the existence of other sharp peaks related to metallic NPs^41^. Finally, the peaks denoted by black stars in Fig. 5B,C were indexed by comparing them to the Cr_2_O_3_ structure reported in the (JCPDS) card 74–0326^42,43^. From the diffraction pattern, it has been discovered that as the Cr ratio rises, these peaks become more intense but are still small due to the presence of sharp peaks associated with the high ratio of Ni nanoparticles. By using XRD measurements, the carbonization of the produced nanofibers was confirmed. However, the lack of other crystalline or amorphous Pt phases indicates that Pt is merged into the FCC Ni phase, presenting a true alloy at every particle size.

The peak width at half maximum (β) was accustomed to evaluate the crystallite size (D) by using the following Debby–Scherer formula^44^:

1 $$D=\frac{K\lambda }{\beta cos\theta }$$

where k = 0.9, λ is the x-ray wavelength (0.154056 nm), and θ is the Bragg angle. The crystallite size was calculated and listed in Table 2. Due to the high temperature used during the calcination procedure, it is possible that the MNPs agglomerated with one another and increased the predicted crystallite extent of the processed samples. The produced alloys’ predicted crystallite extent is considerably greater than the commercially available Pt. The table below shows that the criteria for the crystal lattice’s values have also very slightly changed.

**Table 2:** Calculated SA values from the XRD outcomes.

Sample	Peak position (2θ)	(FWHM) (β)	D_avg_ (nm) scherer	SA (m^2^/gm)	(a) Lattice parameter (nm)
C1	43.98	0.417	20.48	25.22	0.3562
C2	44.041	0.553	15.46	37.44	0.3557
C3	44.013	0.431	19.87	33.36	0.3559
Commercial Pt/AC			19.87	85.7	

The surface area (SA) is defined as the total surface area of a solid material per unit of mass, and is dependent on the size of the particles, as well as on the structure and porosity of the material. The SA values were calculated using the subsequent formula^45,46^:

2 $$SA= \frac{6000}{D\rho }$$

where D is the average size of the crystalline realms in nm and ρ is the prepared samples’ density. Table 2 also includes a list of the SA values determined by XRD for the catalysts C1, C2, and C3. The calculated SA values for the produced electrocatalysts are higher than those for the Pd–Co–Mo alloy heated to 900 °C but lower than that of the marketable Pt catalyst, as listed in Table 2^45^. For Ni based catalysts, the 900 °C calcination temperature might be excessively high, leading to atom accumulation and large grain size.

### Electro-oxidation study

#### Surface activation

**Figure 6:**
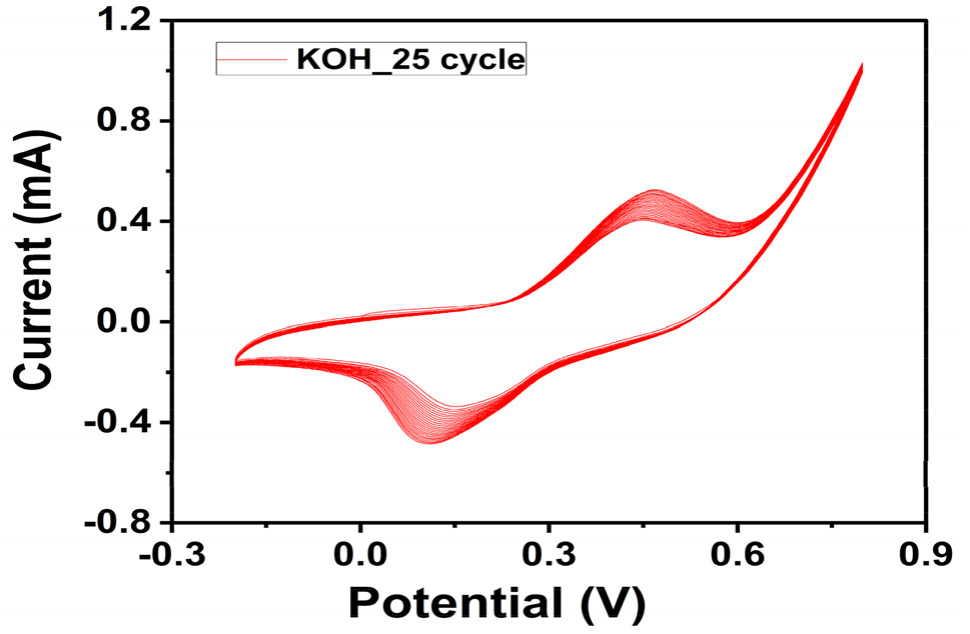
Consecutive cyclic voltammogram of Ni_11_Pt_3_Cr_6%_/CNFs (C3) in 1 M KOH solution at 100 mV/s SR.

The prepared samples were activated using a typical three-electrode cyclic voltammetry system in a 1.0 M KOH to generate a NiOOH layer on the catalyst surface. Figure 6 shows the CV diagram of sample C3 (Ni_11_Pt_3_Cr_6%_/CNFs) nanofiber formulations as an example in 1.0 M KOH solution. Polarization was started by a potential (− 0.2–0.8 V) scanning at a SR of 100 mV/s (vs. Ag/AgCl as RE). Ni Activation can be explained as follow^47,48^:

1 $$Ni{\left(OH\right)}_{2} + {OH}^{-} \leftrightarrow NiOOH + {H}_{2}O + {e}^{-}$$

When the number of potential sweeps is increased, it is possible to gradually develop a thicker NiOOH layer corresponding to the Ni (OH)_2_/NiOOH transition leading to an increase in the current density values.

#### Sample composition effect

**Figure 7:**
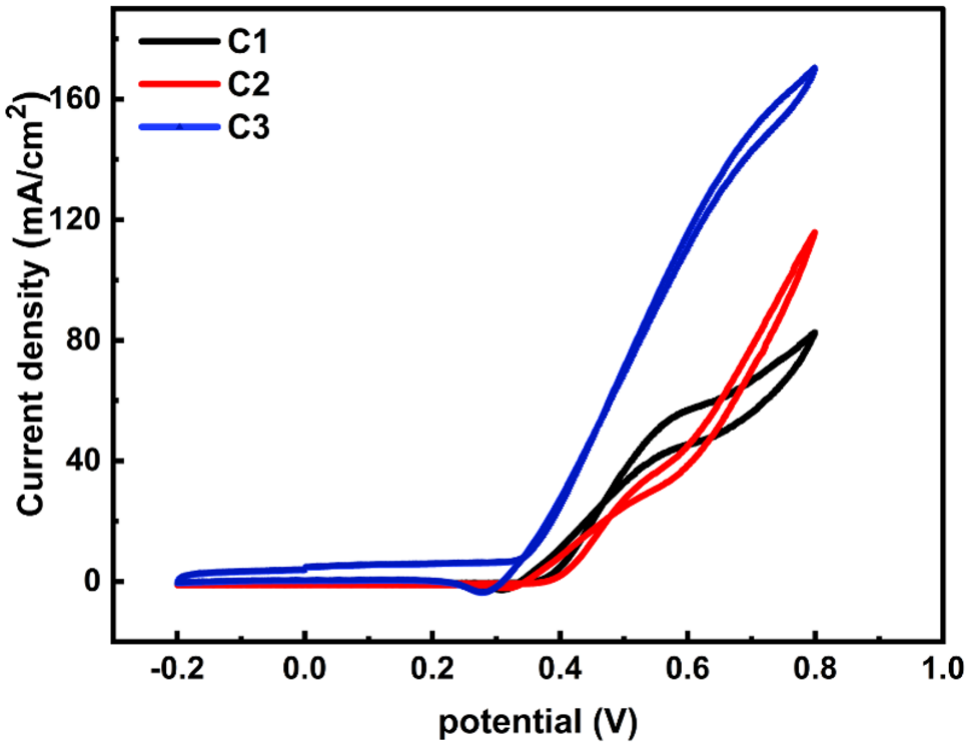
Electric potential dependence of current density for C1, C2, and C3 prepared samples.

Sequential cyclic voltammetry of C1, C2, and C3 using three electrodes, an electrochemical (EC) cell with an Ag/AgCl as (RE), a GC covered with the produced electrocatalyst as (WE), and a platinum wire as (CE). Figure 7 illustrates the impact of the Cr addition on the EC activities for 2.0 M methanol oxidation in a 1.0 M KOH solution at 24 °C and 100 mV/s SR. It is crucial to note that a large current density is produced when the Cr ratio is increased from 0 to 6%. The EC activity, represented in current values, is significantly increased by the addition of Cr. For C1, C2, and C3, the extension of the oxidation peaks caused the current density (J_PE_) values to rise from 73.3 to 107.4 and 170.3 mA/cm^2^. The oxidation peak’s current density (J_P_) for samples C1, C2, and C3 is 51.61 mA/cm^2^ at 0.58 V, 43.3 mA/cm^2^ at 0.55 V, and 139.67 mA/cm^2^ at 0.70 V, respectively, as Ni% decreases from 17 to 14% and 11%. The results reveal the higher electrocatalytic activity of the Ni–Cr–Pt towards methanol oxidation due to the significant synergy effect of Ni and Cr. Hence, the presence of Cr_2_O_3_ nanoparticles promotes the formation of the Ni_III_/Ni_II_ redox pair, which improves catalytic activity and mechanical stability^16–49^.

#### Electrolyte concentration effect

**Figure 8:**
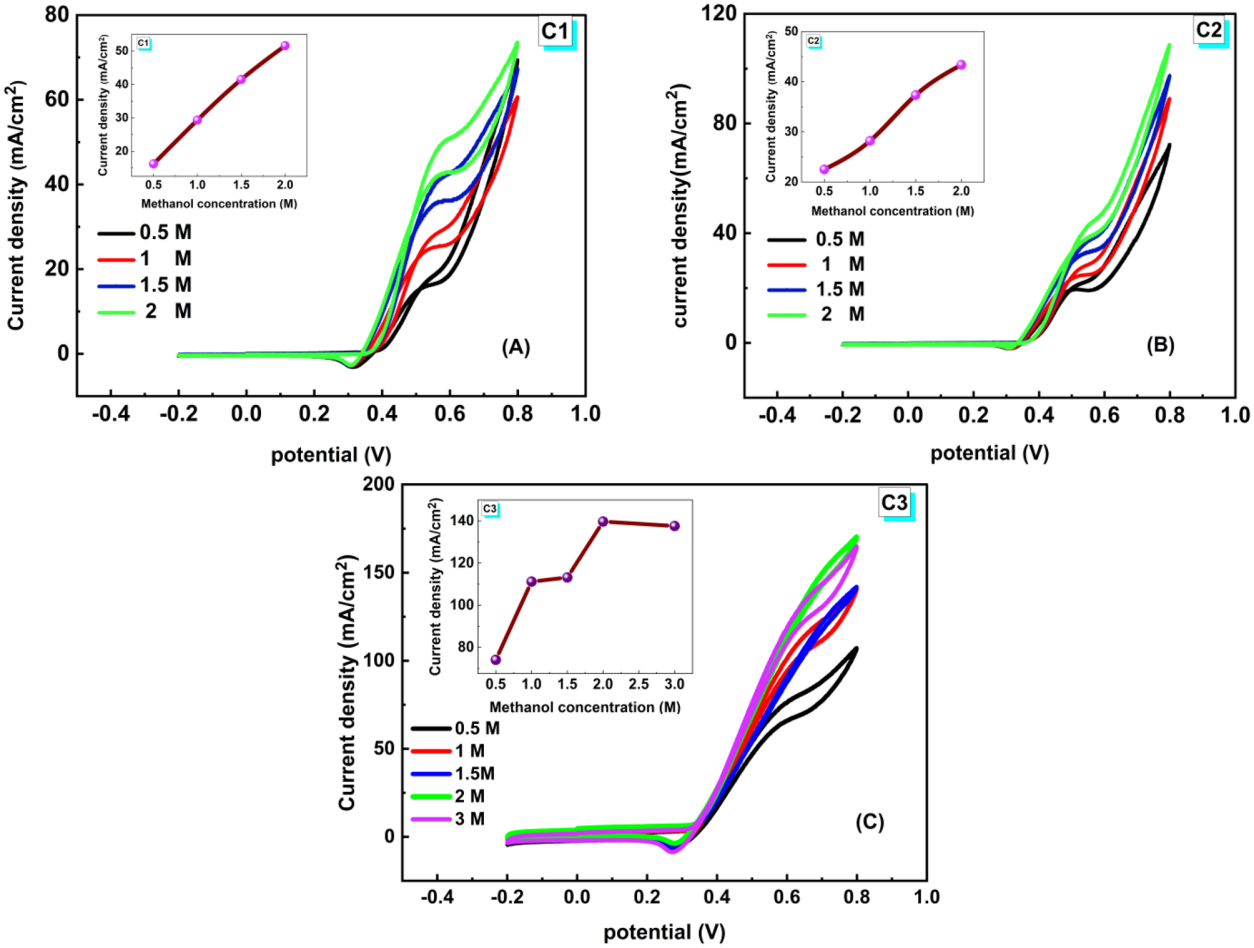
Electrocatalytic activity of the catalysts C1, C2, and C3 in 1.0 M KOH in the existence of 0.5, 1.0, 1.5, and 2 M of methanol, SR 100 mVs^−1^. Inset: the relationship between the peak current and concentrations of methanol.

Figure 8 displays the CV for the different samples in methanol with 100 mV/s SR and an applied potential range of − 0.2–0.8 V. The oxidation peak rises and shifts towards a positive potential as the concentration of methanol increases, forming the basis of the methanol electrooxidation process, as shown in Fig. 8A,B,C, which shows the relationship between the current density and potential in methanol concentrations of 0.5, 1, 1.5, and 2 M for the samples C1, C2, and up to 3 M for C3. Figure 8A for C1 shows that the current densities (J_P_) of the oxidation peaks increase with increasing methanol concentration, from 16.22 mA/cm^2^ at 0.5077 V to 51.61 mA/cm^2^ at 0.5843 V. The JPE value also rose from 69.03 to 73.3 mA/cm2 at 0.8 V as the concentration of methanol increased from 0.5 to 2 M. According to the data presented above, the highest current density value is found at 2 M methanol concentration. For C2, Fig. 8B shows how J_P_ grows with rising methanol concentrations and shifts to an upper potential location. The improvement of the oxidation peaks' current densities (J_P_) is observed in their values, which are 22.50 mA/cm^2^ at 0.5351 V, 28.20 mA/cm^2^ at 0.5407 V, 37.32 mA/cm^2^ at 0.5524 V, and 43.36 mA/cm^2^ at 0.5526 V for 0.5 M, 1.0 M, 1.5 M and 2.0 M of methanol, respectively.

Furthermore, at 0.8 V, the current density of the oxidation peaks extension increased from 70.92 to 107.4 mA/cm^2^. This demonstrates how to sample C2’s EC activity for methanol electrooxidation, which improves with methanol concentration. The methanol electrooxidation process is the next dissociation step based on the adsorption of reactants and intermediates^27^. As shown in Fig. 8C for sample C3, the oxidation peak current densities increased as methanol concentrations increased, from 74.05 mA/cm^2^ at 0.6214 V to 139.67 mA/cm^2^ at 0.6995 V. Increasing the methanol concentration from 0.5 to 3 M, J_PE_ increased from 106.21 to 164.9 mA/cm^2^ at 0.8 V with an increase in concentration, but at 1 M and 1.5 M, the same value of current density was remarked. For C3 also, it can be noticed that, at 3 M methanol, both JP and JPE values are approximately equal or slightly less than those at 2 M. The inset in Fig. 8A,B,C explains the relation between the concentration and the maximum current density. In C3, when adding the methanol up to 2 M, the J_P_ value reaches 139.67 mA/cm^2^. The higher electrocatalytic activity of the ternary metallic catalyst NiPtCr/CNFs towards methanol oxidation is ascribed to the significant synergy effect of Ni and Cr. In addition, Cr can decrease the poisoning of Pt by CO by providing an oxygen group on its surface. The electro-catalytic parameters extracted from the CV data are summarized in Table 3.

**Table 3:** Summary of the electrochemical parameters of the three catalysts.

Samples	Parameters					Tafel slope (mV/Dec)
	Methanol concentration (M)	J_PE_ (mA/cm^2^)	Current oxidation peak J_P_ (mA/cm^2^)	Oxidation potential E_P_ (V)	Onset potential OX (V)	
C1	0.5	69.03	16.22	0.508	0.403	53.3
	1.0	59.61	27.42	0.545	0.386	
	1.5	67.03	42.20	0.571	0.378	
	2.0	73.30	51.61	0.584	0.378	
C2	0.5	70.92	22.50	0.535	0.403	39.7
	1.0	88.70	28.20	0.541	0.401	
	1.5	96.77	37.32	0.552	0.401	
	2.0	107.40	43.36	0.553	0.402	
C3	0.5	106.21	74.05	0.621	0.355	66.2
	1.0	140.32	116.45	0.663	0.346	
	1.5	141.61	121.19	0.697	0.355	
	2.0	170.31	139.67	0.6995	0.352	

J_PE_ is the current density values of the extension peak at potential 0.8 V.

#### Onset potential

Onset potential is a meaningful metric used to assess EC activity. This represents the possibility of the existence of reaction pathways in which all reaction steps have negative free energies. In other words, the electrode overpotential is indicated by the onset potential. In general, a more negative onset potential indicates higher activity and a lower overpotential, which then leads to an increase in over-cell potential^13–50^. Figure S3 shows the relationship between the methanol concentration and the onset potential for the prepared samples. From this figure, it is easy to observe that sample C3 has a small onset potential toward a negative position, which indicates that C3 shows high activity and less overpotential. The right electrocatalyst can lower the onset potential, making it easier for the reaction to occur. This is because electrocatalyst can facilitate the transfer of electrons between the reactants and electrode, reducing the energy required for the reaction to take place. This may be the case of Cr addition to Ni-Pt where Cr reduced the resistance as noticed from EIS measurements and reduced the onset potential. Also, it has been found that the onset potential decreases with decreasing lattice parameter for Bi and Pb series catalysts. The dependence of onset potential and lattice parameter showed a different tendency for Ir and Rh series catalysts, that is, an initial increase and then a decrease. This dependence may be due to the different steric locations of the active center corresponding to the lattice parameters^51^.

#### Influence of scan rate (SR)

**Figure 9:**
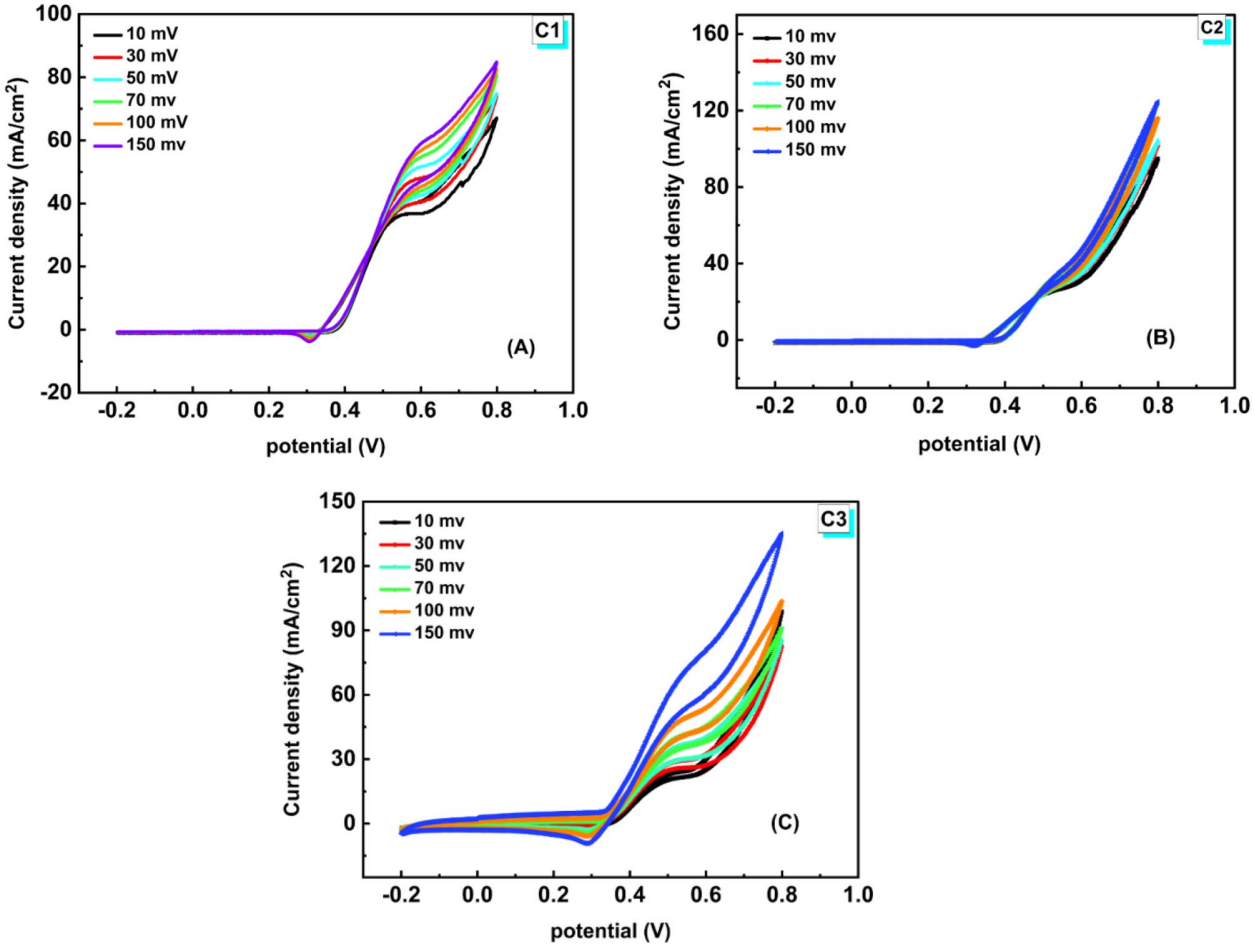
CVs of the investigated NFs (C1, C2, & C3) in the solution of 1.0 M KOH containing 2.0 M methanol at various SRs (10–150 mV/s).

The experiment's scan rate determines how quickly the applied voltage is scanned. Higher currents are seen as a result of faster SRs since they cause the diffusion layer’s size to shrink. The Randles–Sevcik equation (Eq. 3) explains how the peak current i_p_ increases linearly with the square root of the scan rate (V s^-1^) for electrochemically reversible electron transfer processes involving freely diffusing redox species.

3 $${i}_{p}=0.446nFA{C}_{o}{\left(\frac{nF\gamma {D}_{o}}{RT}\right)}^{1/2}$$

where n is the number of the electrons transferred in the redox event, A (cm^2^) is the surface area of the electrode (usually treated as the geometric surface area), *D*_*o*_ (cm^2^ s^−1^) is the diffusion coefficient of the oxidized analyte, and C_0_ (mol cm^−3^) is the analyte bulk concentration. The Randles–Sevcik equation can be used to calculate diffusion coefficients^52^. Figure 9A-C shows cyclic voltammograms of 2 M of methanol at C1, C2, ad C3 catalysts in 1.0 M KOH solution at various SRs (10–150 mV/s). The current of the anode rises progressively with rising SRs ranging from 10 to 150 mV, which may be ascribed to fast electron flow at the interface of the electrolyte/electrode.

**Figure 10:**
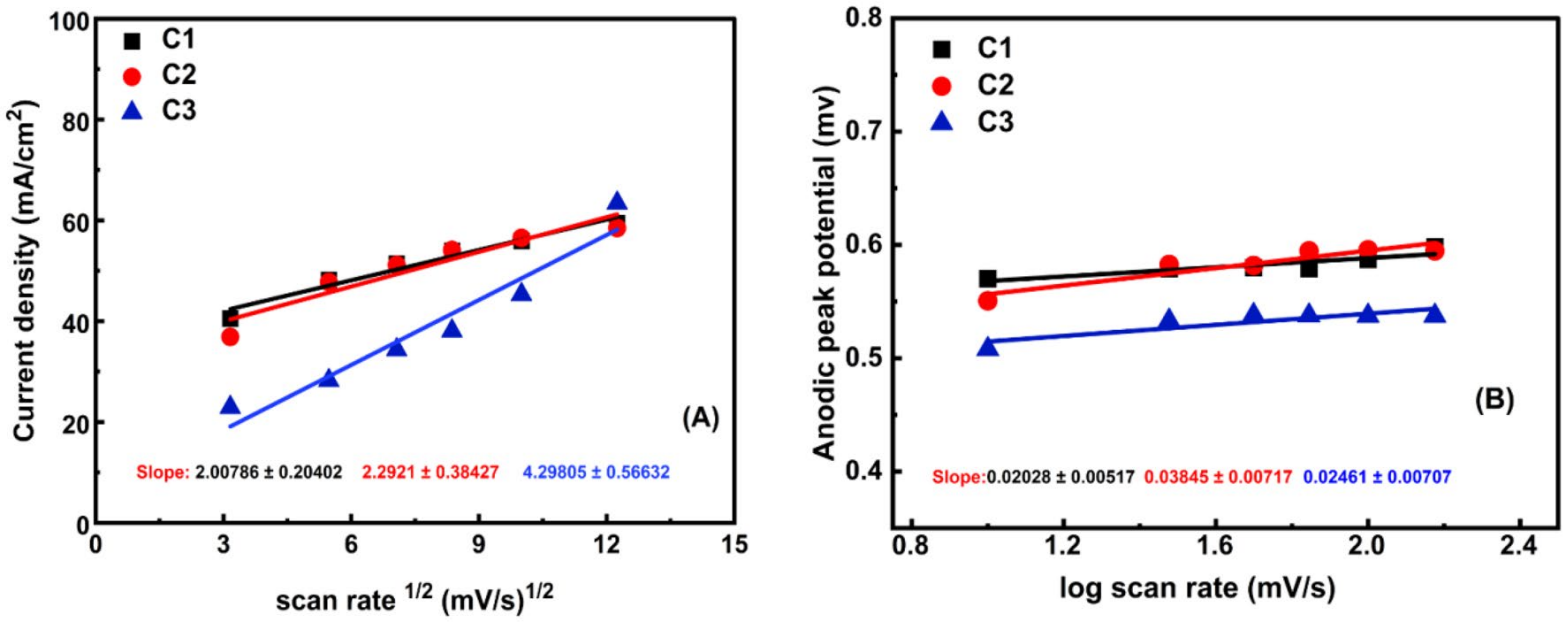
(**A**) Current density against scan rate^1/2^ and (**B**) the logarithm of anodic peak potential versus scan rate1 in the solution of 1.0 M KOH containing 2.0 M methanol on catalysts C1, C2, and C3.

At maximal methanol oxidation, as shown in Fig. 10A, the substantially straight plot of the current density against the square root of the SR supports a diffusion-controlled process. The graph of log (anodic peak potential) as a function of log ((SR)), Fig. 10B, further elucidates the diffusion-based properties. These findings indicate that oxidation is controlled by diffusion^53,54^. As the SR matures, improving methanol kinetic oxidation is referred to as "enhancing oxidation current density". The logarithm of anodic peak potential versus SR is in a linear relationship with the kinetic threshold of the reaction shown in Fig. 10B. The potential's square root determines how tall the peak is. Electrochemical processes that are thermodynamically reversible take place when the anodic peak positions stay constant with the SR^55,56^. As presented in Fig. 10, the electrochemical reaction is not completely reversible, as the cathodic tip potential shifts to a less positive potential while the anodic tip potential becomes more positive as the SR increases. Consequently, as opposed to the reversible state, the anodic–cathodic peak separation becomes greater. With a potential of 0.8 V, the catalysts C1, C2, and C3 now exhibit current densities of (84.84 mA/cm^2^), (124.51 mA/cm^2^), and (135.16 mA/cm^2^), respectively. The greatest current density value belongs to sample C3 (highest Cr ratio, 6%) at 0.8 mV potential compared with C1 and C2. This reflects the synergistic effect of Cr on C3's catalytic activity^57^.

#### Linear sweep voltammetry (LSV) and Tafel slope

**Figure 11:**
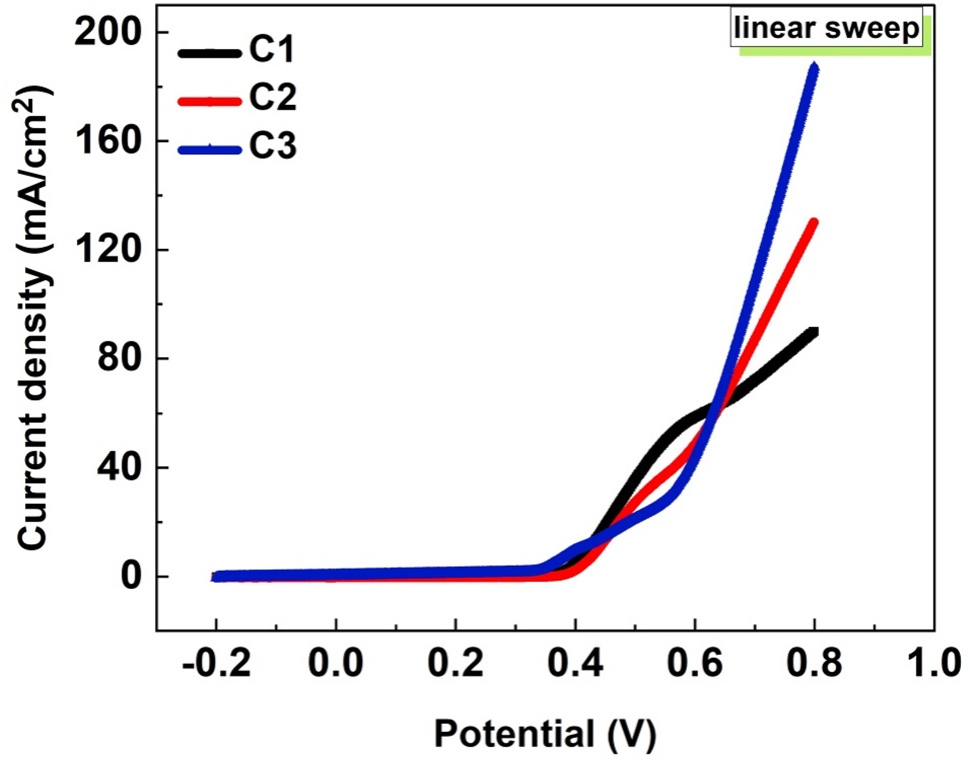
LSV plots of the produced NFs (C1, C2 & C3) in the solution of 1.0 M KOH containing 2.0 M methanol at RT with SR 10 mV/s.

Figure 11 displays the LSV profiles for the investigated NFs (C1, C2, and C3). A three-electrode cell was utilized to perform the LSV curves in methanol (2.0 M)/KOH (1.0 M) at a 10 mV/s scanning rate in a potential window from 0.2 to 0.8 V. At 0.8 V for methanol, the current density grows from 90 to 187.0 mA/cm^2^. When the results of the different samples are compared, C3 has the highest current density value at 800 mV potential compared to C1 and C2.

As shown in Figure S4, the electrochemical reaction kinetics were studied using Tefal curves. The overpotential loss when the operational current density is elevated by 10 times is the physical meaning of the Tafel slope. In line with that, the Tefal slopes of the produced NFs C1, C2, and C3 for methanol are 53.3, 39.7, and 66.2 mV/ Dec, respectively. It is obvious that the Tafel slope of C3 is a bit higher than that of C1 and C2, where C3 has the maximum ratio of Cr. The Tafel slope depends on the electron number involved in the electrode reaction and the charge transfer coefficient. One step with one electron transfer is all there is to a reaction, In brief, any variation in the Tafel slope indicates a change in the mechanism of the electrode reaction. Changes in the Tafel slope, due to changes in the charge transfer coefficient, have effects on the *i*-*E* and logarithmic plots when the exchange current density is assumed to be a constant of *i*_*0*_ = 5 × 10^–7^ A/cm^2^^58^.

#### Study of electrode stability

**Figure 12:**
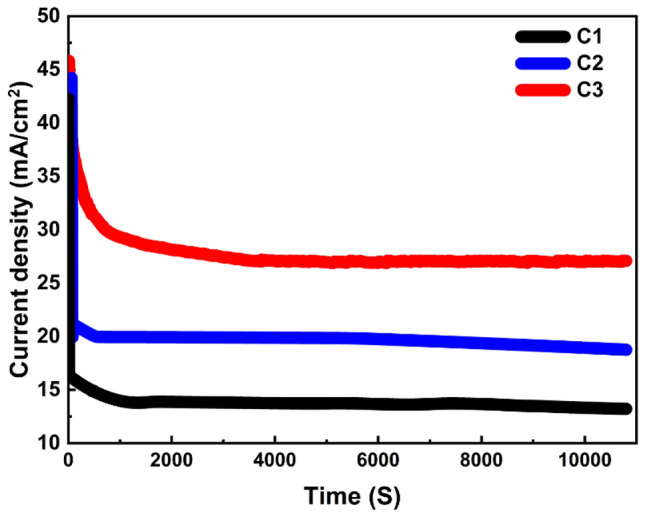
Measurements of chronoamperometry for the sample's current density’s variation over time for C1, C2, and C3 in Methanol.

The chronoamperometric technique is characterized as a successful strategy to examine the prepared catalyst's stability through the stepped potential of the working electrode, and then the current (i) is measured dependently on time (t). Figure 12 displays the long-term stabilities of the investigated catalysts with various Ni/Pt/Cr % ratios as valued by using chronoamperometric measurement over an extended length of time (10,800 S) in KOH solution (1 M concentration) containing 2 M of methanol at 0.8 V potential.

The C3 catalyst shows the highest initial current density (47 mA cm^−2^). The initial spike can be attributed to the higher methanol concentration on the catalyst’s surface at the beginning, and the current stabilizes after some time where the system reaches the equilibrium. It has been detected that all the current–time curves contain two stages. In the first stage of about 50 s, the current beaded rapidly, mostly due to the accumulation of CO-like intermediates on the sample's surface. These adsorbed species may inhabit many of the free active sites of the prepared catalysts, preventing methanol molecules from further adsorption and oxidation. After this stage, the current slowly decreases, and the electrochemical reaction reaches a steady state. This is because of the adsorption of oxygenated poisonous species and their redox reactions maintain relative equilibrium^59,60^. Catalyst C3’s reaction was more stable toward methanol electrooxidation the longer it took to reach a stable output rate, from 50 to 10,800 s. A regulated corrosion process between the electrode and the redox electrolyte can account for this. This can be explained by a controlled corrosion process between the electrode and the redox electrolyte. These results show that the C3 electrode has high chemical stability and a long lifetime as a functioning electro-oxidation electrode, supporting the use of Cr as a co-catalyst to increase the EC of Ni–Pt catalysts.

#### 3.5.8 Electrochemical impedance spectroscopy (EIS)

**Figure 13:**
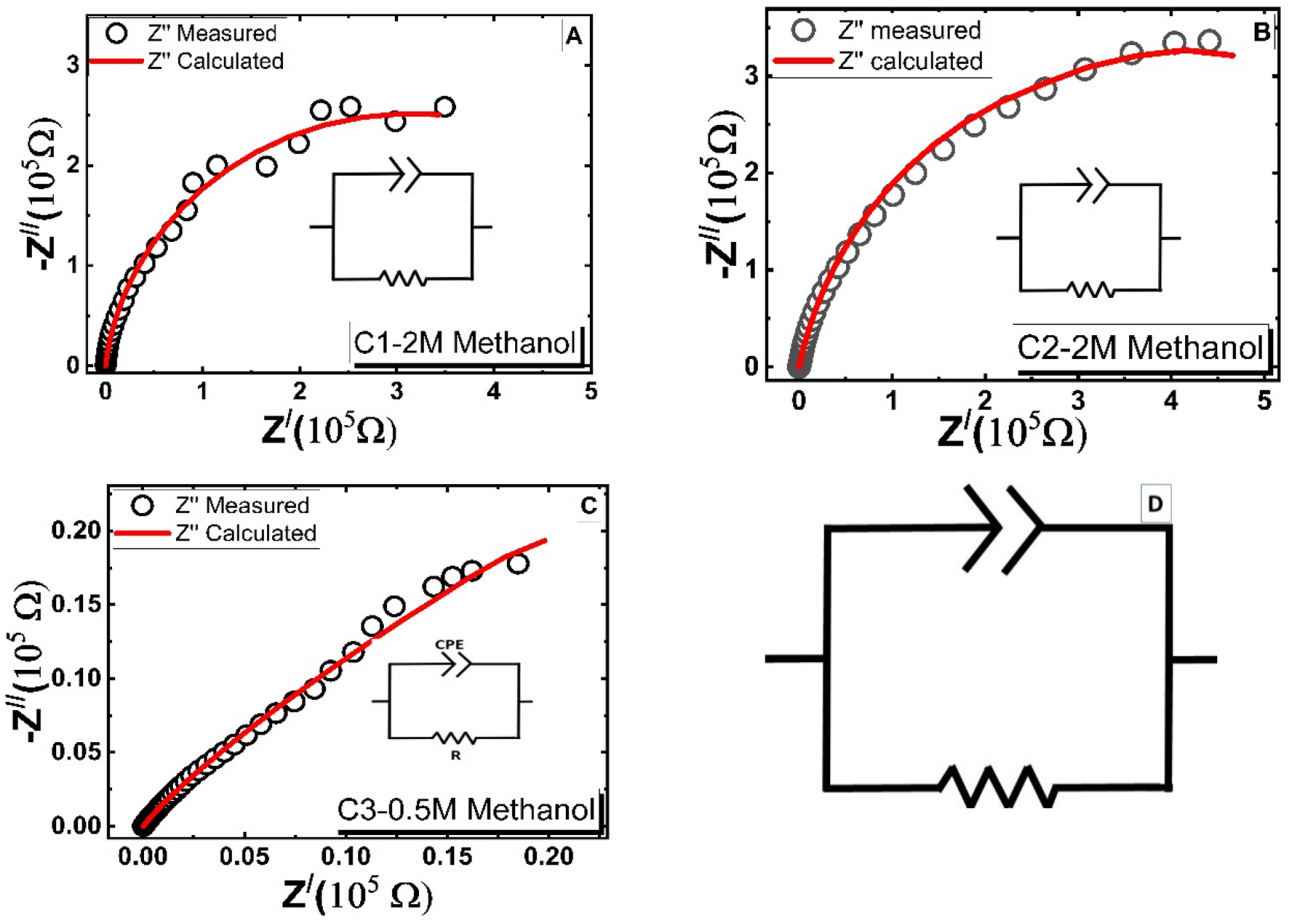
Cole–Cole plot of (**A**) C1, (**B**) C2, and (**C**) C3 investigated samples, and (**D**) equivalent circuit obtained by EIS software.

Charge carrier dynamics has a significant impact on the working electrodes' electrooxidation catalytic efficiency. An electrochemical workstation was used for EIS experiments at RT to look into the charge carrier dynamics of the samples (CH Instruments CHI660E). EIS is a valuable approach for assessing the electrocatalyst's interfacial characteristics^61^. With the C1–C3 electrode submerged in methanol electrolytes, EIS measurements were carried out at 0 V (versus Ag/AgCl) in the frequency range of 0.01–100 kHz. Figure 13A–C demonstrates the Nyquist plots of the investigated nanocatalysts in the solution of 1 M KOH containing 2 M methanol. ZsimDemo software was utilized to fit the measured impedance of C1, C2, and C3 electrodes, and [R CPE] was the equivalent circuit according to the calculated impedance as shown in Fig. 13. Figure 13A shows that the obtained equivalent circuit of the investigated electrodes consists of a constant phase element (CPE-1) connected in parallel with an ohmic resistance (R); this is in good agreement with the results published in the reference^62^. R is the charge transfer resistance of the electrodes, and CPE-1 (also denoted by Y0-CPE) represents the constant phase element of the sample between the electrode and the electrolyte. CPE or Q can be defined as the imperfect capacitor; in other words, it is a wonky capacitor that describes the behavior of a double layer as an imperfect capacitor (electrolyte–electrode or electrode-substrate in our case).

Mathematically, the definition of CPE is like that of a capacitor, as follows:

4 $${Z}_{CPE}=\frac{1}{{Q}_{0}{(j\omega )}^{n}}$$

where n is the frequency power corresponding to the CPE values, its values range from 0 to 1. When n = 1 then it describes an ideal capacitor while n = 0 describes a pure resistance. The observed semicircle for each specimen is a clear indication of the charge transfer resistance at the electrode–electrolyte interface, as it is shown in Fig. 13D. Because it is challenging to identify the precise elements that contribute to impedance, impedance values are calculated by modeling related circuits and fitting the reactions at the interfaces thought to occur in an actual reaction system. The primary reactions of each specimen in accordance with its equivalent circuit can be determined by measuring the impedance value with the lowest estimated error using the fitting approach. The overall impedance values that were determined by fitting the analog circuit are shown in Table 4.

**Table 4:** Impedance values obtained from equivalent circuit fitting.

Sample	Equivalent circuit	CPE ($\mu$ *F*·sec^n^)	Frequency power (n1)	R (kΩ)
C1	[RQ]	9.5 $\pm$ 0.86	0.87 $\pm$ 0.004	$580\pm$ 5.81
C2	[RQ]	9.1 $\pm$ 0.19	0.82 $\pm$ 0.0027	$877\pm$ 8.77
C3	[RQ]	146 $\pm$ 1.79	0.58 $\pm$ 0.0048	169 $\pm$ 6.7

As shown in the table, C1 (580 KΩ) and C2 (877 KΩ electrodes have higher charge transfer resistance values, while the C3 electrode has the lowest charge transfer resistance (169 KΩ), which improves the electrooxidation catalytic process. CPE is an important element in the equivalent semicircle, which represents a part of the impedance and can help explain the reactions of a real system. With CPE, the impedance element may be inferred from the value of the frequency power (n); the closer to 1 and 0.5 and 0 the n value is, the more likely it is to show the characteristics of capacitance's impedance, Warburg impedance, and resistance, respectively^63,64^. As it was mentioned above, CPE represents the constant phase element of the sample between the electrode and the electrolyte. For the electrodes C1 and C2, n1 is closer to the value 1, which means that the impedance is characterized by a capacitor, while for electrode C3, n1 is close to 0.5, which reveals the characteristics of Warburg impedance. This indicates the reduction of the capacitance, which in turn improves the process of the charge transfer. The capacity of an electric system needed to store electric charge is indicated by capacitance. The building of electric charge occurs more quickly, and the transfer of electric charge becomes more difficult as capacitance increases^65–68^.

**Figure 14:**
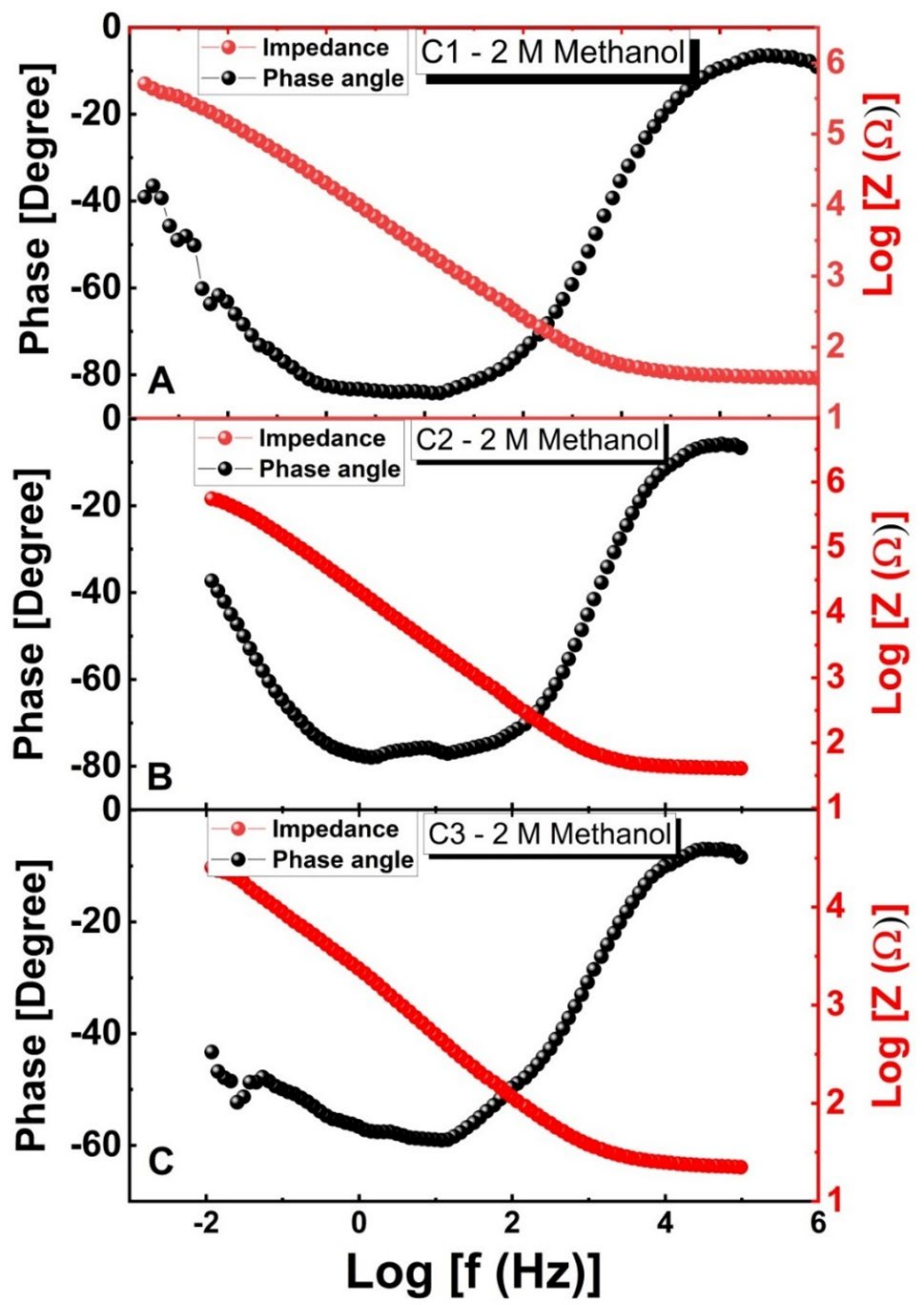
(**A**–**C**). Bode plots for electrodes C1, C2, and C3 in methanol at the optimized concentrations and 0 V (vs. Ag/AgCl).

Bode plots for all electrodes using methanol at 0 V (vs. Ag/AgCl at RT) are shown in Fig. 14A–C. Figure 14 shows the relationship between the phase and the frequency logarithms, as well as the relationship between the log of the total impedance Z and the log of the frequency. The charge transfer resistance causes a resistive regime at low frequencies, while the electrode’s double-layer capacitance causes a very tiny capacitive contribution at high frequencies, as seen by the plot of Log (Z) against Log (f)^69^. With an increasing Cr ratio, at the electrolyte/electrode contacts, charge recombination has been significantly reduced. A kinetically straightforward electrooxidation procedure, an increase in ionic conductivity, and electrolyte diffusion throughout sample C3 are also included in this. As a consequence, this electrode outperformed the other electrodes in terms of electrooxidation catalytic performance. All samples are collected at the lower phase angle of the NiPtCrO/CNFs coated GC electrode (40° at 0.1 Hz). Given that ideal capacitive systems should have phase angles of − 90°, this may indicate that the NiPtCrO/CNFs nanocatalyst exhibits less capacitive behavior^70^.

## Conclusions

Electrospinning is a quick, low-cost, and efficient method for creating carbon nanofibers that are doped with Cr atoms and include NiPt nanoparticles. Remarkably, calcinations of the manufactured electrospun nanofiber mats at 900 °C for 7 h in an argon environment led to the creation of well-morphed carbon nanofibers that also showed NiPtCrO nanoparticles. The chemical composition, architectures, morphologies, and electrochemical assets were studied using different analysis techniques. The calcination method of the electrospun nanofibers diminishes the fiber width from 250–520 to 216–380 nm for catalysts with various concentrations of Cr (0–6%). The mapping analyses showed that Ni, Pt, and Cr were distributed uniformly across the nanofibers' surface. From X-ray results, the NiCr-Pt/CNFs crystalize in a face-centered cubic (FCC) structure, with a crystallite size ranging from 15.46 to 20.48 nm with various concentrations of Cr. Voltammetric analysis, electrode stability, electrolyte content, (SR) impact, Tafel slope, and EIS spectroscopy were utilized to inspect the electrocatalytic activities of the fabricated catalysts for methanol oxidation in KOH. The electrooxidation and electrode stability could be successfully increased by adding Cr as a co-catalyst with Ni–Pt. Additionally, sample C3’s current density at 2 M electrolyte concentration was 139.67 mA/cm^2^. This sample has a negligible tendency to start negatively; this show C3 to be very active and to have a lower overpotential. It also has the lowest electrolyte resistance and charge transfer resistance among the other electrodes. The greatest current density value belongs to sample C3 (highest Cr ratio, 6%) at 0.8 mv potential comparing with C1, and C2. This reflects the synergistic effect of Cr on C3’s catalytic activity.

## Supplementary Information

Supplementary Information.

## Data Availability

The datasets used and/or analysed during the current study available from the corresponding author on reasonable request.
