# Supplementary material for: Methanol electro oxidation on Ni–Pt–CrO/CNFs composite: morphology, structural, and electrochemical characterization
Source: Sci Rep. 2023 Mar 24;13:4870. doi: 10.1038/s41598-023-31940-x (PMC10039033; doi:10.1038/s41598-023-31940-x)
Supplement: Supplementary file 1 — Supplementary Information. [file 41598_2023_31940_MOESM1_ESM.docx]

**Methanol Electro Oxidation on Ni-Pt-CrO/CNFs Composite:Morphology,Structural, and Electrochemical Characterization**

E.E. Abdel-Hady^1^, Ahmed Gamal^2^, Hany Hamdy^2^, Mohamed Shaban^2, 3^, M.O. Abdel-Hamed^1^, Mahmoud A. Mohamed^1^, [Wael M. Mohammed](https://sciprofiles.com/profile/1409217)^1^

^1^Physics Department, Faculty of Science, Minia University, Minia P.O. Box 61519, Egypt

^2^Nanophotonics and Applications (NPA) Lab, Department of Physics, Faculty of Science, Beni-Suef University, Beni-Suef 62514, Egypt

^3^Physics Department, Faculty of Science, Islamic University of Madinah, P.O. Box 170, Madinah 42351, Saudi Arabia

*****Correspondence:  [esamhady@mu.edu.eg](mailto:%20esamhady@mu.edu.eg) & [esamhady@yahoo.com](mailto:esamhady@yahoo.com); Tel.: +201096669635

**
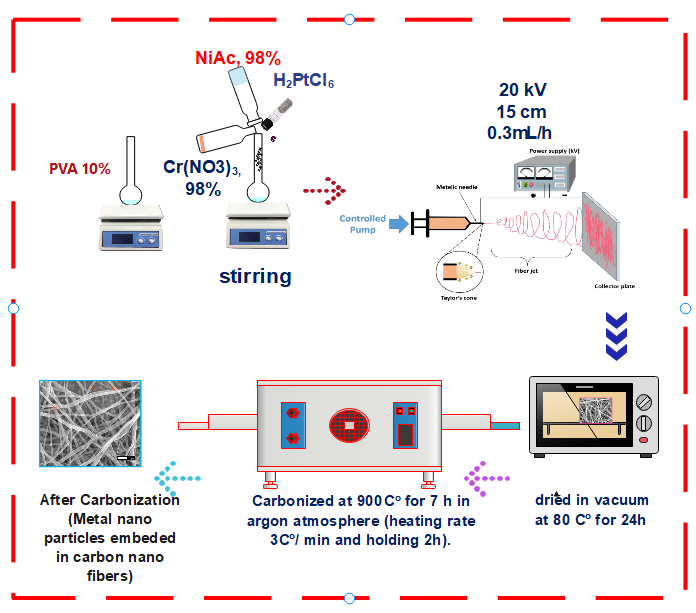
**

Figure S1: Preparation steps for Ni Pt Cr /CNFs


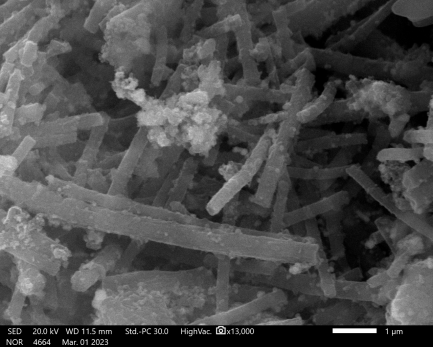

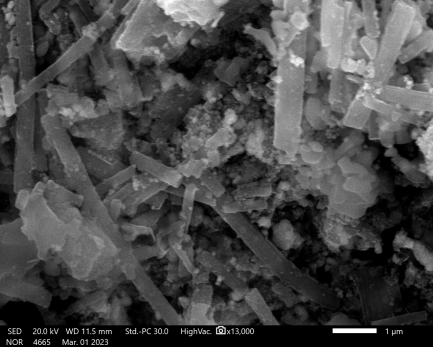

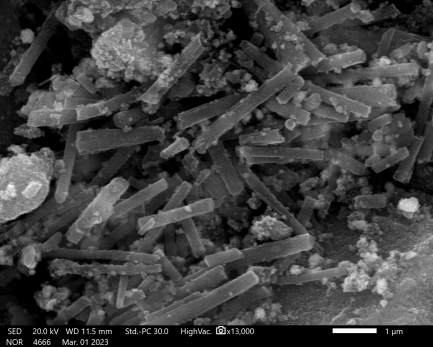


**C3**

**C2**

**C1**

Figure S2: SEM images of the prepared catalysts C1, C2, and C3 after

oxidation process.


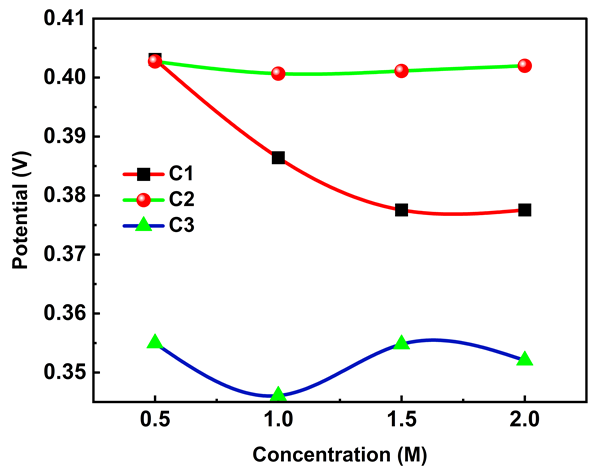


Figure S3: Onset potential versus methanol concentration (0.5 M, 1.0M, 1.5 M & 2 M)

for the prepared catalysts C1, C2, and C3


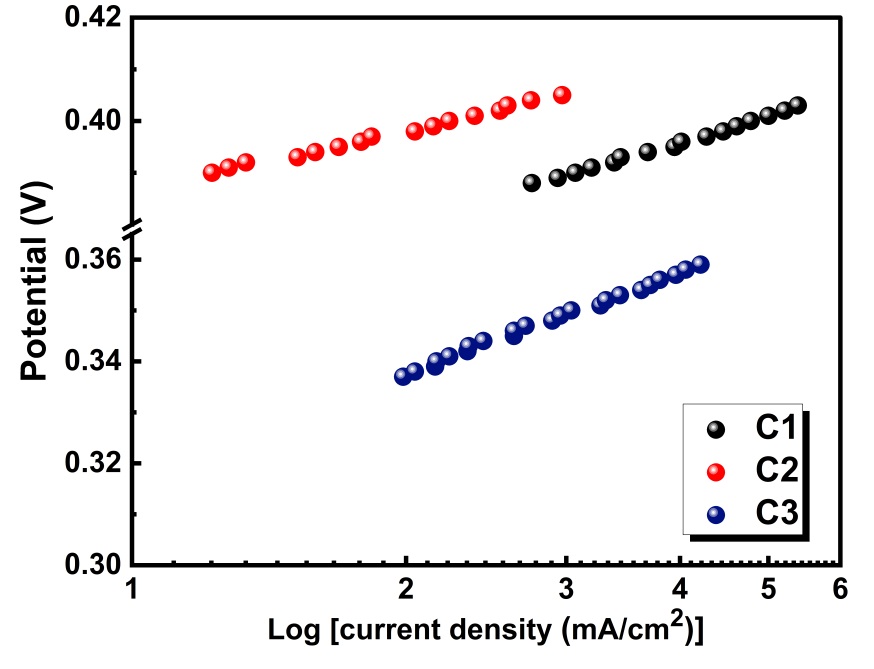


Figure S4: Tafel slope for the prepared catalysts C1, C2, and C3.
